# Altered RNA metabolism due to a homozygous *RBM7* mutation in a patient with spinal motor neuropathy

**DOI:** 10.1093/hmg/ddw149

**Published:** 2016-05-18

**Authors:** Michele Giunta, Shimon Edvardson, Yaobo Xu, Markus Schuelke, Aurora Gomez-Duran, Veronika Boczonadi, Orly Elpeleg, Juliane S. Müller, Rita Horvath

**Affiliations:** 1 Institute of Genetic Medicine, Newcastle University, Central Parkway, NE1 3BZ, Newcastle upon Tyne, UK; 2 The Monique and Jacques Roboh Department of Genetic Research, Hadassah, Hebrew University Medical Center, 91120 Jerusalem, Israel; 3 Department of Neuropediatrics and NeuroCure Clinical Research Center, Charité-Universitätsmedizin, Charitéplatz 1, 10117 Berlin, Germany

## Abstract

The exosome complex is the most important RNA processing machinery within the cell. Mutations in its subunits *EXOSC8* and *EXOSC3* cause pontocerebellar hypoplasia, spinal muscular atrophy (SMA) and central nervous system demyelination. We present a patient with SMA-like phenotype carrying a homozygous mutation in *RBM7*—a subunit of the nuclear exosome targeting (NEXT) complex—which is known to bind and carry specific subtypes of coding and non-coding RNAs to the exosome. The NEXT complex with other protein complexes is responsible for the substrate specificity of the exosome. We performed RNA-sequencing (RNA-seq) analysis on primary fibroblasts of patients with mutations in *EXOSC8* and *RBM7* and gene knock-down experiments using zebrafish as a model system. RNA-seq analysis identified significantly altered expression of 62 transcripts shared by the two patient cell lines. Knock-down of *rbm7*, *exosc8* and *exosc3* in zebrafish showed a common pattern of defects in motor neurons and cerebellum. Our data indicate that impaired RNA metabolism may underlie the clinical phenotype by fine tuning gene expression which is essential for correct neuronal differentiation.

## Introduction

The exosome complex is a major component in degradation, maturation and quality control of almost any type of RNA within the cell, and therefore plays a major role in gene expression regulation (1). The exosome is a large multi subunit complex whose functions are highly conserved throughout all forms of life (2). In eukaryotes it is composed of nine subunits, six of them forming the core of the protein complex through which the RNA will pass in a 3′–5′ direction (Rrp41/EXOSC4, Rrp42/EXOSC7, Rrp43/EXOSC8, Rrp45/EXOSC9, Rrp46/EXOSC5 and Mtr3/EXOSC6) and three of them forming the cap of the exosome (Rrp40/EXOSC3, Csl4/EXOSC1 and Rrp4/EXOSC2) with RNA binding properties (3). As previously mentioned, the exosome complex performs a number of functions related to gene expression regulation through RNA decay (4). Other than being engaged in 3′–5′ degradation of normal mRNAs, it degrades mRNA which contain AU-rich elements (AREs) (5). The exosome also degrades defective mRNAs through different pathways: the ‘nonsense-mediated’ decay (NMD) pathway, which degrades mRNAs with a premature stop codon (6), the ‘non-stop’ decay pathway (NSD) degrades mRNAs that lack a termination codon (7), and the ‘no-go’ decay pathway (NGD) which targets mRNAs on which translation has stopped (8). More recently it has been shown that the exosome complex is involved in the metabolism of many types of non-coding RNAs (9). The exosome complex deals with such a high variety of substrates through the interaction with different co-factors which bind specific subtypes of RNA (10). One of the main cofactors is the trimeric Trf4/5p-Air1/2p-Mtr4p polyadenylation (TRAMP) complex. The two catalytic activities of the TRAMP complex are carried out by TRf4-Air2—a poly(A)polymerase sub-complex - and helicase Mtr4 (11). MTR4 co-purifies with the exosome complex in yeast (12). Human homologs of the TRAMP components, in addition to hMTR4, might be hTRF4-1 (POLS) and hTRF4-2 (PAPD5) (12) and ZCCHC7 (Air2) (13) although experimental evidence is lacking.

The human MTR4 together with an RNA-binding protein (RBM7) and a Zn-knuckle protein (ZCCHC8) forms another trimeric complex, the nuclear exosome targeting (NEXT) complex (12). NEXT is present in the nucleoplasm to assist the exosome-driven degradation of PROMoter uPstream Transcripts (12) and other non-coding RNAs (14).

The superkiller (SKI) complex co-operates with the exosome in the cytoplasm. The SKI complex in yeast is a hetero-tetramer formed by one copy of Ski2 and Ski3 and two copies of Ski8 (15) and seems to be involved in degradation of defective mRNAs through the NMD, NSD, and NGD pathways (10). It is not fully understood how the SKI complex works in humans as the protein which mediates the interaction between the exosome and SKI (Ski7p) in yeast has not been identified in humans. However, a paralog of Ski7p, Hbs1p, which does have an ortholog in humans might take over the role of Ski7p (10).

Correct processing of mRNAs as well as non-coding RNAs is known to be fundamental for normal cellular functions (16). Wan et al. (17) showed for the first time that mutations in the exosome complex can cause human disease, when they reported mutations in the *EXOSC3* sub-unit of the exosome in pontocerebellar hypoplasia and spinal muscular atrophy (SMA) (PCH1), accounting for about 40% of cases worldwide (18). Our group subsequently identified mutations in *EXOSC8* which are responsible for the development of overlapping symptoms of PCH, SMA and central nervous system (CNS) demyelination (19). In the last 2 years several other studies indicated that mutations in *EXOSC3* result in a more variable clinical presentation, suggesting that exosome complex dysfunction is a major cause of severe childhood onset complex inherited neurological disorders. More recently, also mutations in *EXOSC2* found in two unrelated German families were shown to cause neurodevelopmental defects such as mild cerebellar atrophy and cerebellar hypoplasia, diffuse dysmyelination, hearing loss, mild intellectual disability, facial anomalies and premature ageing (20).

In this study, we present a patient with a SMA-like phenotype with a homozygous mutation in *RBM7*, a co-factor of the exosome complex (c.236C > G; p.Pro79Arg). In order to understand the different roles of different components of the exosome complex and its co-factors in neurodevelopment we performed analysis in human cells carrying mutations in exosomal proteins and in *rbm7, exosc3* and *exosc8* knock-down zebrafish (*Danio rerio)*.

## Results

### Patient

The patient is the youngest of seven siblings to consanguineous Palestinian parents. Family history is negative for similarly affected children. Pregnancy, delivery and perinatal course were uneventful except for breech presentation which necessitated caesarean section. Initial concerns were raised around one month of age as hypotonia with poor sucking and failure to thrive were observed. No developmental regression or cognitive difficulties were noted but gross motor abilities plateaued around 1 year of age when unsupported brief sitting was achieved. At this time the patient was brought to our clinic where examination revealed an alert child with no dysmorphic features, but cachexia (weight 6.025 kg, <10 percentile; height 70.7 cm, <10 percentile; head circumference 43.2 cm, <3 percentile) with reduced muscle mass and decreased tendon reflexes. No tongue fasciculations, or poly-mini-myocloni were noted. Muscle weakness, both proximal and distal was apparent (3/5 for most muscle groups tested). Respiratory difficulties were evident from a few months of age onwards and mechanical ventilation became necessary during inter-current illnesses due to hypercapnia and hypoxemia, though weaning was successful several times. During the last episode of respiratory decompensation at age of 28 months the patient died.

**Figure 1.**
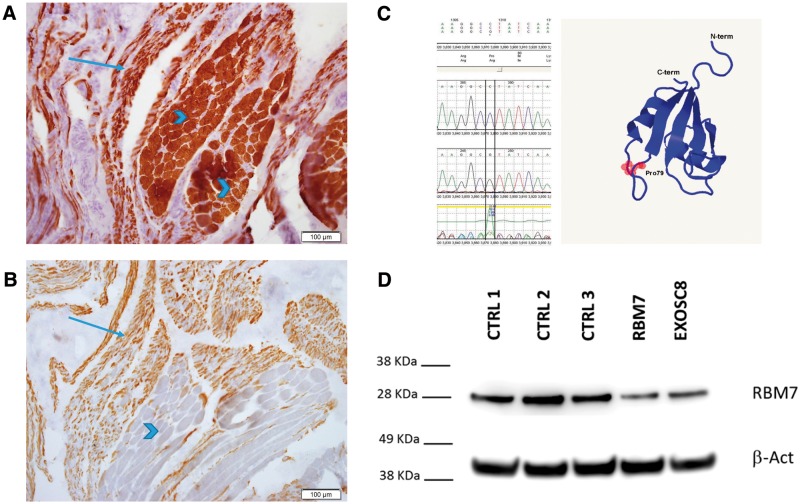
Frozen sections stained with immunohistochemical stains for slow- **(A)** and fast-myosin **(B)** display striated muscle tissue with large group atrophy, including atrophic myofibers of both types, alongside groups of hypertrophic myofibers, most of them type 1. The c.236C > G; p.Pro79Arg mutation identified by Sanger sequencing is predicted to be pathogenic by Mutation Taster (58); **(C)**. The mutated amino acid is located in the RRM domain just before the last β strand forming a four stranded Antiparallel β-sheet. The 3D model was built using Phyre2 according to the structure presented by Hrossova et al., 2015. Immunoblotting of fibroblasts of the patient with *RBM7* mutation and *EXOSC8* mutant fibroblasts showed reduced RBM7 protein levels in both patients compared with three control fibroblast lines **(D).**

Brain MRI was normal. Muscle biopsy detected including fibre type grouping of small and hypertrophic fibres, compatible with SMA as shown in Figure 1A and B. No other significant alterations were evident on H&E, GTC, ATPase9.4, ATPase4.3, NADH, SDH/COX, PAS, PAS + D and ORO stains. Paraffin embedded sections displayed sheets of foamy macrophages (CD68-immunopositive), and only few myofibers, consistent with macrophagic myofasciitis. electromyography/nerve conduction velocity (NCV) was also compatible with SMA showing very low CMAPs with chronic early and late re-innervation with otherwise normal NCV motor and sensory studies. *SMN1* analysis showed two normal copies. Routine laboratory results including serum creatine kinase and cardiac echo were normal.

## Genetic Analysis

### Whole exome sequencing analysis detected a homozygous mutation in *RBM7*

Whole exome sequencing (WES) of the patient yielded 58.1 million mapped reads with a mean coverage of X86. Following alignment and variant calling, we performed a series of filtering steps. These included removing variants called <×8, were off-target, synonymous, heterozygous, MAF > 1% at ExAC (Exome Aggregation Consortium, Cambridge, MA, (URL: http://exac.broadinstitute.org)) or MAF > 4% at the Hadassah in-house database (∼800 ethnic matched exome analyses). 31 variants survived this analysis but only 11 remained (Supplementary Material, Table S1) after removal predicted benign variants (Mutation Taster). Using Sanger sequencing, we then confirmed segregation within the family for the *RBM7* and the *SNX15* variants (Supplementary Material, Table S2). The human RBM7 gene is located on chromosome 11, has 5 exons and encodes 5 different isoforms. The longest isoform codes for a 266 amino acid protein (NM_016090.3, AF156098.1). The mutation c.236C > G; p.Pro79Arg is located within the highly conserved RNA Recognition Motif (RRM) domain (14) and is predicted to be pathogenic, affecting the structure of the binding domain (Fig. 1C) as well as the splice site (according to MutationTaster), decreasing the stability of the protein structure (MuPro—http://www.ics.uci.edu/∼baldig/mutation.html; Confidence Score: −0.068480655 and Confidence Score: −0.644794635393117). In silico analysis with PROVEAN (http://provean.jcvi.org/index.php) also predicted the mutation to be deleterious with a score of −4.49. Align-GVGD (http://agvgd.iarc.fr/agvgd_input.php) scored it Class C65 (most likely to interfere with protein functions). This variant has not been detected to date in any of the large databases. Another homozygous variant was detected in the patient in the *SNX15* gene. This variant has been reported with very low frequency previously (Supplementary Material, Table S1). Mutations in *SNX15*, a novel sorting nexin involved in protein trafficking (21) have not been associated with human disease to date, and recent data suggests that this protein is important for cell surface recycling of APP and Aβ generation (22). Given its function, it is more unlikely to be causative for the SMA-like phenotype, however we cannot exclude completely that it contributes to the clinical manifestation of the disease. Extensive search for similar patients with mutations in *RBM7* could not be identified despite we screened 24 further patients with SMA phenotype, where SMN1 testing was negative and searched over 1000 exome sequencing analysis of patients with neuromuscular or neurogenetic disease, suggesting that RBM7 is a rare cause of SMA-like disease.

### Immunoblotting

Significantly reduced level of the RBM7 protein (63% reduction) was detected in patient fibroblasts compared with three healthy controls, and RBM7 was also reduced in another fibroblast cell line carrying a pathogenic mutation in *EXOSC8* (19) (Fig. 1D). Immunoblotting for SNX15 detected normal protein levels (data not shown). Considering the function of the two proteins in the cell, the similarity of clinical features of this patient and the *EXOSC8* patients, and the reduced protein levels of RBM7 but not SNX15 in patient cells, we conclude that the *RBM7* variant is more likely to be the disease causing mutation in this patient.

### RNA-seq analysis

**Figure 2.**
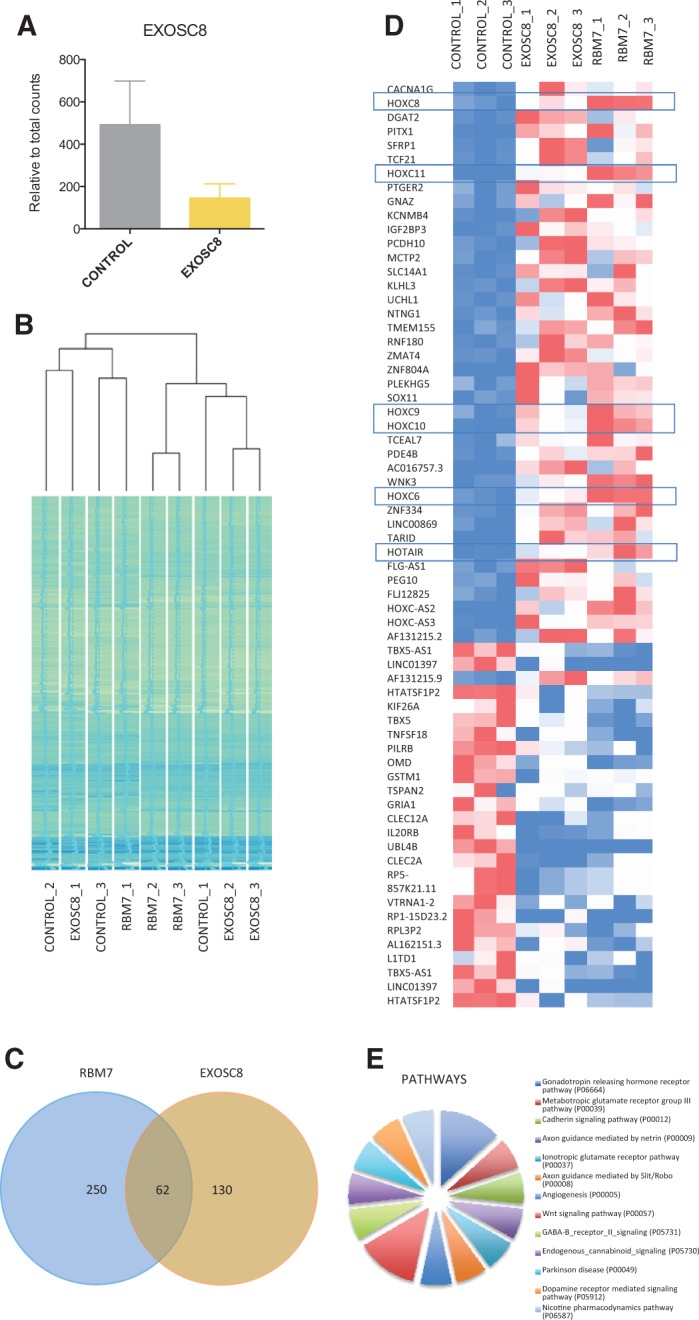
Transcriptomic characterization of fibroblasts carrying mutations in *RBM7* and *EXOSC8*. **(A)** Levels of EXOSC8 transcripts of patient with the c.5C > T, p.Ala2Val *EXOSC8* mutation and control fibroblasts. Relative corrected counts are represented. **(B)** Hierarchical clustering dendrogram for RNA-seq data for all covered genes for the 3 biological triplicates of each cell line. The range of colours between blue and green represent the number of corrected counts. **(C)** Venn diagram showing the share genes between the differentially expressed genes of both comparisons. **(D)** Heat map of the distribution of the corrected values of the shared genes. HOX genes are highlighted. Red represented higher counts, white middle and blue lower counts, across the comparisons. **(E)** Pathway enrichment analysis performed in the 62 shared genes by Panther pathway analysis.

For each sample, at least 13 million read pairs were mapped to the transcriptome with their strands matched to the annotated transcripts. As expected, there was a reduction in the levels of the EXOSC8 transcript in the *EXOSC8* cells (Fig. 2A), compared with the control, but not in the *RBM7* cells (data not shown). Hierarchical representation of the samples showed how most of the triplicates clustered together (Fig. 2B). RNA-seq analysis showed a > -fold change in gene expression of several protein coding genes and non-coding RNAs, 62 of which are being shared between *EXOSC8* and *RBM7* mutant primary fibroblasts (Fig. 2C and D, Supplementary Material, Table S4). Interestingly, pathway analysis of the shared RNAs showed pathways related to the nervous system (Fig. 2E).

### Gene expression of *HOXC* genes was altered in human fibroblast with *EXOSC8* and *RBM7* mutations

**Figure 3.**
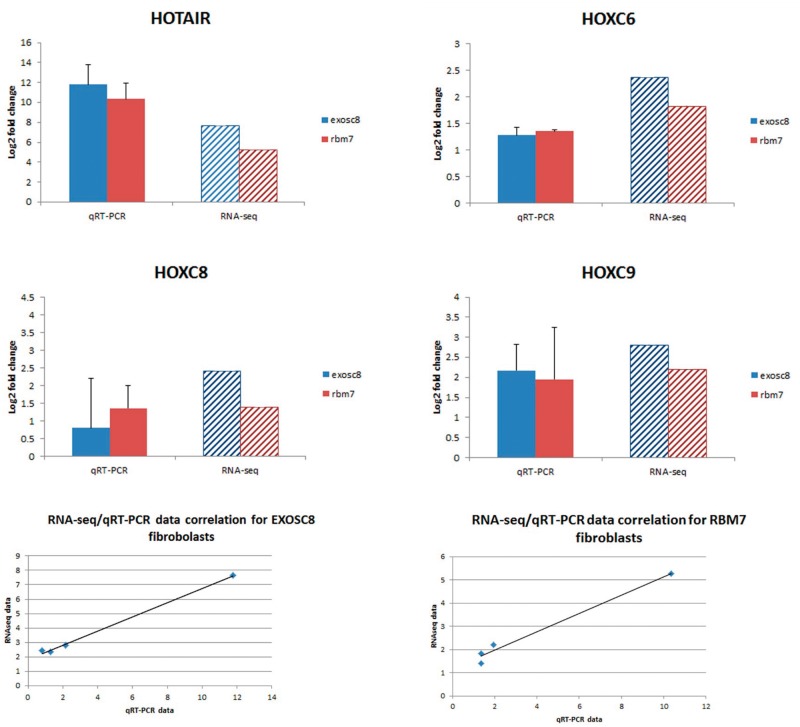
Gene expression analysis of four genes through qRT-PCR confirmed RNA-seq data. RNA-seq data and qRT-PCR show a high correlation. The qRT-PCR data show relative expression of target genes in RBM7 and EXOSC8 mutant fibroblasts compared with control fibroblasts. Analysis was repeated on three biological replicates.

In order to verify RNA-seq data we performed qRT-PCR analysis of four transcripts which were upregulated in the RNA-seq analysis. Correlation analysis between RNA-seq and qRT-PCR data of *HOTAIR, HOXC6, HOXC8, HOXC9* confirmed a high degree of correlation between the two sets of data (RBM7: *R*^2 ^= 0.9806; EXOSC8: *R*^2 ^= 0.9973, Fig. 3). We detected no significant changes in genes associated with motor neuron and Purkinje cell (PC) dysfunction or myelination that could be expected from the phenotype of the RBM7 and EXOSC8 patients. This could be explained by tissue specific differences, as these genes might not be expressed in fibroblasts.

### Abnormalities in morphant zebrafish resemble the clinical phenotype of patients with exosomal protein deficiencies

In order to verify RNA-seq data from human fibroblasts, we decided to use zebrafish as a model system for exosomal protein deficiencies. Zebrafish is considered an excellent animal model for investigation of neurodevelopmental disorders (23) and has been previously used to study cerebellar development (24), hindbrain (25) as well as motor neurons (26). The lightly pigmented *golden* strain and *islet1*:GFP transgenic zebrafish were chosen to perform downregulation of *rbm7*, *exosc8* and *exosc3.* MOs for *exosc3* and *exosc8* have been previously reported and respective morphants were phenotypically characterized (17,19). Our database search (ENSDARG00000020841.7, NM_199925.1) identified a single *rbm7* gene in the zebrafish genome encoding a 252 amino acid protein located on chromosome 18. Although the overall homology between the human and zebrafish RBM7 protein is relatively low (41% identical and 55% similar protein sequences) and also the mutated amino acid is not conserved between the two species (it is substituted with a glutamine in zebrafish), if only the highly conserved region of the RRM (the first 94 amino acids in human, the first 93 in zebrafish) is considered, the degree of homology is much higher (14) (69.5% identity and 84% similarity, Supplementary Material, Figure S1).

**Figure 4.**
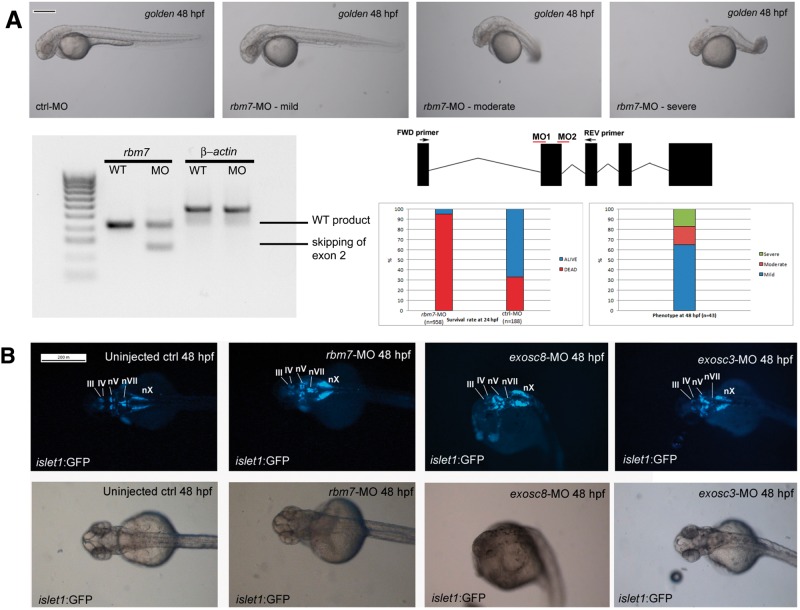
Morphological defects caused by injection of rbm7 MO **(A)**. Mild phenotype fish show slight developmental delay, and shorter body length. Rostrally the head is smaller than control fish while caudally the head looks slightly swollen. Body shape in the moderate phenotype looks compromised and head oedema increased, sporadically we could observe heart oedema as well. Body shape of the severe fish is completely disrupted, swelling of the head is more severe, and we detected smaller forehead and eyes compared with control fish (Scale bar = 200 μm). On the bottom left: RT-PCR of zebrafish cDNA shows efficacy of morpholino against In1-Ex2 boundary. Position of morpholinos is explained in the graph on the right. Below: graphs showing mortality and phenotype rates at 24 hpf. **(B)** Hindbrain defects in morphant fish. Cranial motor neurons nuclei are well defined in 5 nuclei in wild type fish: Oculomotor neurons (III), Trochlear neurons (IV), Trigeminal neurons (V), Facial neurons (VII), Vagal neurons (X). *rbm7*-MO seems to have a milder effect on cranial motor-neurons development and to affect predominantly vagal nucleus (nX) which result shorter than controls. Hindbrain structures in exosc8-MO are more generally affected the moderate phenotype fish. Only nX is partially preserved. Exosc3-MO affects predominantly nVII (facial neurons) (Scale bar = 200 μm).

To knock down *rbm7*, we designed an antisense MO to target the intron1-exon2 boundary (Fig. 4A), which caused a range of different phenotypes and morphant fish were classified in three classes according to the severity of the phenotype (mild, moderate, severe). Injection of *rbm7*-MO caused a series of developmental defects. Even the mildly affected fish were unable to swim away normally upon touch stimulation compared with the control fish (supplementary videos). The body shape was slightly shorter and sporadically brain oedema could be observed. The moderately affected fish had a curved body shape, smaller head and eyes compared with control fish. Heart and brain oedema were frequently observed. In fish with severe phenotype the body shape was completely altered, the tail was absent, and the head and eyes were smaller. Co-injection of *rbm7*-MO (2.2 ng) with p53-MO (5 ng) in order to reduce apoptosis did not significantly reduce the high mortality rate within the first 24 h, which seems to happen after 12 hpf. The number of dead fish in the control MO injected group was not much different from control un-injected fish, indicating that the mortality was caused by the *rbm7* MO. We designed a second morpholino to target exon2-intron2 boundary, and could obtain a very similar phenotype, indicating that the phenotype is specific for *rbm7* knock down

.

### Hindbrain nuclei were altered in *islet1*:GFP morphant zebrafish

Downregulation of all three genes led to brain abnormalities in *islet1:GFP* fish (Fig. 4B), resembling what previously observed by us in an *exosc8-*MO zebrafish model (19) and by others in a zebrafish model of SMA (27). Down-regulation of *rbm7* has a milder effect on more rostral cranial motoneurons, affecting specifically the nuclei of the vagal brachimotor neurons (X) even in the mild phenotype. Knock down of *exosc3* affects most severely the migrating brachimotor facial neurons (VII) while other areas are relatively less compromised. Disruption of hindbrain organization in *exosc8-*MO-treated fish is more generalized showing scattered structures without any recognizable anatomical area in moderately affected. A little effect was observed on hindbrain development in *exosc8-*MO mildly affected fish, but the brain phenotype becomes more prominent in parallel with the severity of the changes in overall body morphology. Knock-down of the three genes shows interestingly that, although overall the anatomical structures of the hindbrain were disrupted in all three morphants, knock-down of different genes caused more severe impairment in different hindbrain areas.

### Imaging of motor neurons and PCs in *golden* morphant zebrafish detected characteristic abnormalities resembling PCH1

**Figure 5.**
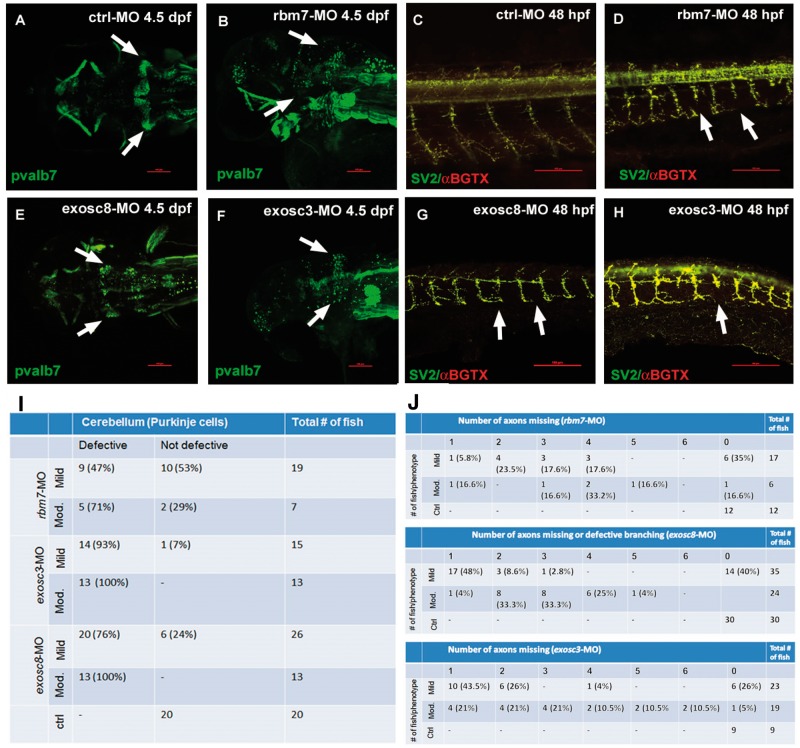
Confocal microscopy analysis of PCs **(A,B,E,F)** and neuromuscular junctions **(C,D,G,H)** in zebrafish upon injection of three different MOs. PCs fail to differentiate in rbm7-MO (B), exosc8-MO (E) and exosc3-MO (F) zebrafish compared with ctrl-MO fish (A). The percentage of fish with cerebellar defects varies significantly between morphants, reflecting what observed in patients. Motor axon growth is defective in all three morphants (D,G,H); arrows point at abnormally short motor axons. Interestingly, only in exosc8-MO the motor neuron axon branches in close proximity of the spinal cord instead of more ventrally as in the ctrl-MO fish (G). Scale bar = 100 μm. We show the quantity and respective percentage of fish with cerebellar defects **(I)**. Axonal defects in different morphant and phenotypical classes (only mild and moderate phenotypes were considered for this analysis) **(J)**. The number of defective structures increases with the severity of the phenotype.

We performed whole mount immunohistochemistry in zebrafish embryos and larvae at 4.5 dpf and 48 hpf to investigate the role of *rbm7, exosc8* and *exosc3* in the development of the peripheral and CNS in order to find similarities with the human phenotype. We used antibodies against Pvalb7 (a kind gift of Professor Masahiko Hibi, Nagoya University, Japan), a marker of PCs in the cerebellum and SV2 antibody and α-bungarotoxin which bind, respectively to presynaptic vesicles and AChRs allowing the visualization of neuromuscular junction (Fig. 5).

We observed a similar pattern of cerebellar disruption upon injection of all three MOs but in different proportions (Fig. 5A and B, E and F, I). Only fish with a mild and moderate phenotype were considered for this experiment, because of non-specific changes are frequent in severely altered embryos. The structure of PC layer showed scattered cells instead of a typical wing-shaped structure, in all 3 MO treated fish. About 75–100% of the *exosc3*-MO and *exosc8*-MO and <50% of *rbm7*-MO-treated fish showed defective structures (Fig. 5I).

Staining of neuromuscular junctions showed overlap of presynaptic and postsynaptic structures and defective growth of motor neuron axons in all three morphants (Fig. 5C and D, G and H, 5J). Many of the fish have one or more axons truncated at about 1/3 to 1/2 of the thickness of the body (Fig. 5J). Interestingly, only *exosc8*-MO fish showed defective branching of motoneurons which divide in two branches after about one-third of the total length (Fig. 5G). The reduced length of the motor neuron axons was statistically significant (Supplementary Material, Figure S2).

### Gene expression analysis in zebrafish

Based on the increased expression of the *HOXC* genes in human cells and their role in neuronal development we performed gene expression analysis by qRT-PCR of HOX genes in *rbm7*, *exosc8* and *exosc3* morphant zebrafish. We did not detect any significant difference compared with control fish (data not shown). Although some of the analysed HOX genes were up or down-regulated upon gene knock down, the difference was always <2-fold change.

**Figure 6.**
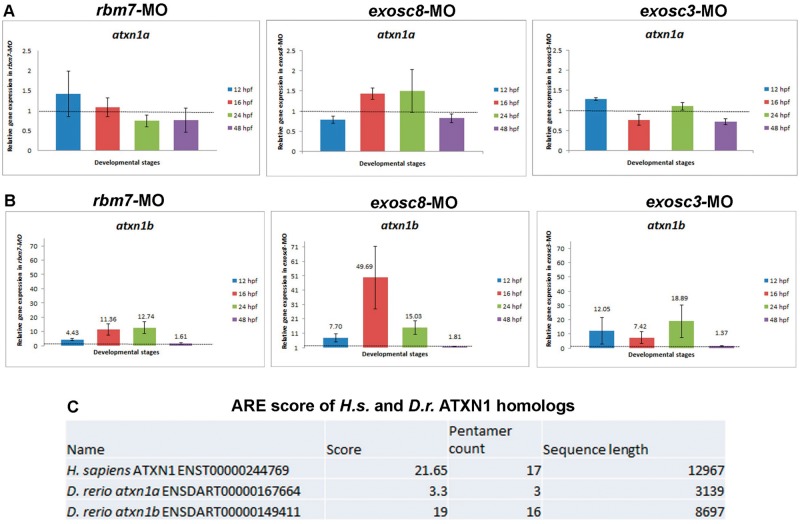
Gene expression analysis of *atxn1a* and *atxn1b* in zebrafish upon knock down of *rbm7, exosc8 and exosc3*. Knock down of all three genes did not affect expression of *atxn1a* **(A)** while expression of *atxn1b* was greatly affected by defective functionality of the exosome complex **(B)**. Error bars indicate SD qRT-PCR analysis repeated three times on different biological samples. Gene expression data are in agreement with the AU-content of *atxn1a* and a*txn1b* **(C)**. Human *ATXN1* has an AU-content similar to zebrafish *atxn1b*.

Interestingly, expression of *atxn1b* (but not *atxn1a)* resulted to be several folds upregulated upon knock down of all three genes in zebrafish. In silico analysis of the sequences of *atxn1a* and *atxn1b* with ARE score (http://arescore.dkfz.de/arescore.pl) searching for the level of AU content showed that *atxn1b* has a very high ARE score, similar to humans, while *atxn1a* is not AU rich (Fig. 6). One of the main function of the exosome complex is the degradation of ARE genes, which may explain why only the ARE *atxn1b* was increased in our zebrafish models of exosomal protein deficiencies.

## Discussion

Here we report a homozygous missense change in the *RBM7* gene as likely cause of the disease in a patient with spinal motor neuropathy, similar to SMA. This finding further emphasises the important role of RNA metabolism in spinal motor neurons. The patient developed hypotonia and failure to thrive at one month of age, followed by delayed motor development, muscle weakness and respiratory problems with normal cognition, and died of respiratory failure at 28 months of age. Electrophysiological examination and muscle histology were all suggestive of SMA. Exome sequencing identified a homozygous missense mutation in *RBM7*, and our investigations support the pathogenic role of this mutation in the human disease. The mutation c.236C > G; p.Pro79Arg is located within the highly conserved RRM domain (14) and is predicted to affect RNA binding. It resulted in severely reduced levels of the RBM7 protein to 37% of the levels in control cells. We also detected lower RBM7 in another patient with EXOSC8 deficiency, suggesting a tightly regulated interaction within the exosome and related complexes. In support of this, we also detected lower EXOSC3 levels in EXOSC8 deficiency (19).

RBM7 is a subunit of the NEXT complex, a co-factor of the exosome. It is an RNA binding protein known to be involved in RNA processing in eukaryotes (14,28). Its RRM domain is comprised within the first 94 amino acids in humans and binds with high specificity to U-rich or AREs (14).

It has been previously suggested, that *EXOSC3*, *EXOSC8* and *RBM7* have different roles in the eukaryotes’ exosome. *EXOSC3*/Rrp40 have RNA-binding properties, containing one Ribosomal protein S1-like RNA-binding domain (S1) and one K homology RNA-binding domain (KH) binding domain (10). *EXOSC8*/Rrp43 has a Pleckstrin homology domain, but it is not clear if it has phosphorolytic activities or if instead this subunit only has a structural function (29). Finally, *RBM7* has a RRM domain which is involved in both RNA binding (14) and splicing (28).

Given the involvement of RBM7 in the cellular RNA metabolism, we studied the global RNA levels in patient and control cells by RNA-Seq analysis. We detected differential expression of 200–300 genes in each cell lines, and 62 genes were the same in both RBM7 and EXOSC8 mutant cells. Many of these genes were homeobox (HOX) genes and non-coding RNAs. HOX genes are a class of highly conserved transcription factors, and they play a very important role during embryonal development and mutations in these genes have very variable clinical presentations (30,37). HOX genes are divided in four clusters (A–D) involved in body axis definition (31), in the development of the limbs (32) as well as the central and peripheral nerves (33). It is uncertain, however, whether the changes in the expression of *HOX* genes relate to our patient phenotype. We also detected increased levels of a special long non-coding RNA transcript, *HOTAIR* in both *RBM7* (5.26 log2 fold change) and *EXOSC8* (7.64 log2 fold change). *HOTAIR* is a long non-coding RNA which was shown to be involved in transcriptional and epigenetic regulation (34), as well as in post-translational modifications (35) of several other genes, including ataxin (*ATXN1*) (35). *HOTAIR* is located in a transgenic region in the *HOXC* locus and co-transcribes with these genes (36) and also silences the expression of the HOXD genes (36). This pattern is clearly seen in our *EXOSC8* mutant fibroblasts, where the increase in HOTAIR is associated with reduction of *HOXD10*, *HOXD11* and *HOXD13* of respectively −6.67, −5.37 and −4.10 Log2 fold change. These *HOXD* genes have been shown to be involved in neurodevelopment in humans (37) and mice (38).

To study the role of RBM7 deficiency *in vivo* we generated zebrafish models of *rbm7*, but also of *exosc8* and *exosc3* deficiencies, the phenotype of these animals closely resembled that of patients carrying autosomal recessive mutations in these genes. Interestingly, we were able to observe a similar pattern of neuronal defects as previously observed in a zebrafish model of SMA both in peripheral (39) and cranial nerves (27). Knock-down of *rbm7*, *exosc8* and *exosc3* also caused defective development of cerebellar structures. Disruption of the structure of the PC layer in our models is keeping with what has been previously shown by *in situ* hybridisation in a zebrafish PCH1 model (17). The cellular structure of the cerebellum and its functions are highly conserved among all vertebrates given that it is necessary to perform motor tasks, integrating sensory and motor information (40). Between 75 and 100% of *exosc3-*MO and *exosc8-*MO fish have defective cerebellum compared with only <50% of the *rbm7-*MO fish (Fig. 4I), indicating a more important role of *exosc3* and *exosc8* in cerebellar development. Similarly, patients with *EXOSC8* and *EXOSC3* mutations have severe pontocerebellar hypoplasia (41) while the *RBM7* mutation causes an SMA-like phenotype.

Based on the observation of increased expression of *HOXC* genes in human fibroblasts we tested whether this is also present in zebrafish models of exosomal protein deficiencies. Although we observed mild changes in the expression of some *hox* genes, none of these changes were statistically significant. No ortholog of HOTAIR has been characterised in zebrafish yet. Based on the observation of defective cerebellar PC differentiation in morphant fish, and on the fact that *ATXN1* is a known ARE gene important for PC survival we decided to investigate further this gene after knock-down of *rbm7*, *exosc8* and *exosc3* in zebrafish. *ATXN1* is known to be linked to the pathogenesis of spinocerebellar ataxia type 1 (42). Extended polyQ stretch within the protein causes toxicity and neurodegeneration of PCs, the nuclei of the brainstem cranial nerves, the inferior olives, and the spinocerebellar tracts (43,44). ATXN1 is important for differentiation of PCs in mouse (45). Neurodegeneration caused by ATXN1 is not only due to the gain of function caused by the polyQ extension. Also overexpression of the wild type gene seems to be toxic in mouse as well as in *Drosophila melanogaster*, indicating that higher levels of normal ATXN1 protein domains (other than the expanded polyQ tract) might also contribute to neurodegeneration (46,47). There are two *ATXN1* paralogues in zebrafish: *atxn1a* and *atxn1b*, both expressed in the cerebellum as early as 28 hpf (48). Increased levels of *atxn1b* mRNA was identified upon knock-down of *rbm7*, *exosc8* and *exosc3* in our zebrafish models, which may have a toxic effect on the development of the cerebellum leading to a defective differentiation of PCs observed in zebrafish at 4.5 dpf. Notably, the percentage of fish with defective cerebellar structure is directly proportional to the level of *atxn1b* in the corresponding morphants. Altered expression of other neuron-specific genes may underlie the degeneration of motor neurons or oligodendroglia. Further studies in neuronal cells will reveal tissue specific factors in RNA metabolism, which may provide further insights to the understanding of neurodegenerative diseases.

## Materials and Methods

### Genetic analysis

Ethical approval and informed consent from the patient’s family has been obtained for the study.

### WES analysis 

Exonic sequences from DNA samples of the patient were enriched with the SureSelect Human All Exon 50 Mb Kit (Agilent Technologies, Santa Clara, CA, USA). Sequences (100-bp paired-end) were generated on a HiSeq2000 (Illumina, San Diego, CA, USA). Read alignment and variant calling were performed with DNAnexus (Palo Alto, CA, USA) using default parameters with the human genome assembly hg19 (GRCh37) as reference. Sequencing of the coding region of RBM7 has been performed by intronic primers (Supplementary Materials). Parental consent was given for genetic studies. The study was performed with the approval of the ethical committees of Hadassah Medical Center and the Israeli Ministry of Health.

### Cell culture

Fibroblasts of one patients carrying *EXOSC8* mutations (patient P3-II:1 in Boczonadi et al. 2014) and of the patient carrying the *RBM7* mutation and three controls were grown in high glucose Dulbecco’s modified Eagle’s medium (DMEM, Sigma, Poole, UK) supplemented with 10% FBS and 1% penicillin/streptomycin.

### Immunoblotting

Aliquots of total protein (20 µg) were loaded on 4–12% SDS-polyacrylamide gel electrophoresis, transferred to a PVDF membrane with an iBlot2 PVDF Mini transfer stack and subsequently probed with a polyclonal antibody recognizing RBM7 (Abcam ab84116, 1:600), SNX15 (Abcam ab172534, 1:500), β-actin (Sigma A1978, 1:2000).

### RNA-seq and bioinformatics analysis

Total RNA was isolated from primary fibroblasts using the mirVana miRNA Isolation Kit (Ambion) and DNAse treated with the DNA-free DNA Removal Kit (Ambion). RNA-seq libraries were prepared using Illumina (Illumina, Inc. CA, U.S.) TruSeq Stranded Total RNA with Ribo-Zero Human kit and were sequenced on an Illumina HiSeq 2500 platform using paired-end protocol. The quality of sequencing reads was firstly checked with FastQC (http://www.bioinformatics.babraham.ac.uk/projects/fastqc/). The 12 bp on the left ends and 4 bp of the right ends of all reads were clipped off with Seqtk (https://github.com/lh3/seqtk) to remove GC-content biased bases. Autoadapt was then used to remove low quality bases (Q < 20) and contaminations from standard Illumina paired-end sequencing adaptors on 3′ ends of reads. Autoadapt (https://github.com/optimuscoprime/autoadapt) uses FastQC to identify the exact sources of contaminations and uses cutadapt (49) to remove them automatically. Poly-N tails were trimmed off from reads with an in house Perl script. Only reads that were at least 20 bp in length after trimming were kept. These high quality reads were then mapped to the human reference genome hg38 with Tophat2 (50). Number of reads mapped to genes were counted using HTSeq-count (51). Differentially expressed genes were then identified with Bionconductor (52) package DESeq2 (53). *P*-values of detected expression changes were corrected with Benjamini & Hochberg algorithm. Genes differentially expressed with *P*-values ≤ 0.05 and fold change ≥ 2 were considered as differentially expressed genes.

### Fish strains and maintenance

We used the *golden* zebrafish strain and the transgenic islet-1:GFP strain (Tg(islet-1:GFP) which expresses GFP in cranial motor neurons under the control of *islet-1* promoter (54). Zebrafish embryos were collected and raised at 28.5 °C in E3 medium using established procedures (55) and staged in hours or days post-fertilization, according to the criteria established by Kimmel et al. (56).

### Antisense morpholino microinjections

We used two previously published translation blocking morpholino oligonucleotides (MOs) (Genetools, LLC) for *exosc3* and *exosc8* knock down (17,19). We designed two new splice blocking morpholinos to knock down *rbm7*, in order to target the intron1-exon2 boundary and the exon2-intron2 boundary. The sequences of the MOs were the following:

SPL *rbm7_In1-Ex2-*MO*:* 5′-ATGGCCCAGCCTAGTGGAAAAAGAA-3′; SPL *rbm7_Ex2-In2*-MO: 5′-ACGCAATAAGGAAAGTCCTACCGGT-3′ AUG *exosc8* MO: 5′-TTTAAAACCAGCCGCCATGATGTTT-3′; AUG *exosc3* MO: 5′- TCCATGATGGAGGAGCGGAAAACAC-3′; CTRL MO: 5′-CCTCTTACCTCAGTTACAATTTATA-3′.

Morpholinos were re-suspended in 1× Danieau solution [0.4 mM MgSO_4_, 58 mM NaCl, 0.7 mM KCl, 5 mM HEPES, 0.6 mM Ca(NO_3_)_2_; pH 7.6] with Phenol Red (56). Embryos were injected up to two cells stage of development with 1.5 ng of *exosc3* MO, 10 ng of *exosc8* MO and 2.2 ng of *rbm7* MO. The Gene Tools standard control MO was used for control injections. The dose of MO was optimized by injecting several doses until a range of different phenotypes was obtained. At least three independent microinjections were performed to study the phenotype caused by each MO. The transgenic islet-1:GFP fish strain was used to study cranial motor neuron abnormalities and the ‘golden’ strain was used for immunostaining and qRT-PCR. Zebrafish of the islet-1:GFP strain were anesthetized with Tricaine solution and phenotyped at 48 hpf to assess brain morphology. Images were captured using a MZ16F fluorescent stereomicroscope (Leica).

### Whole mount antibody immunofluorescence

For whole-mount immunofluorescence staining, ‘golden’ zebrafish embryos and larvae were fixed in 4% paraformaldehyde in phosphate-buffered saline overnight at 4°C and then permeabilized in cold acetone at −20°C. In addition, 4-dpf-old larvae were permeabilized with collagenase A (Roche Diagnostics, 1 mg/ml) for 90 min. Embryos/larvae were blocked in 5% horse serum in phosphate-buffered saline containing 0.1% Tween-20 (PBT). Embryos/larvae were incubated in blocking solution containing primary antibody overnight at 4°C followed by washing several times with PBT and incubation with secondary antibody (1:500 anti-mouse Alexa Fluor 488, Invitrogen). Primary antibodies used in this study were: Parvalbumin7 (1:1000, mouse ascites) was used for PCs staining (kind gift of Prof. Masahiko Hibi, Nagoya University, Japan) as previously described in (57); SV2 (1:200, Developmental Studies Hybridoma Bank, Iowa) was used for staining synaptic vesicles. Acetylcholine receptors were visualized by using Alexa Fluor 594 conjugated α-bungarotoxin (1 μg/ml, Invitrogen). Embryos were imaged in methyl cellulose using a Nikon A1R confocal. Z-stack images were generated by scanning through the whole body with a 10× objective and then images manipulated to have the best resolution with NIS-element AR 3.2 64 bit software.

### Image analysis

For measuring motor neuron axon length, a total number of five fish with a mild phenotype were randomly chosen from each morphant group and shorter axons were measured. Measuring was performed with NIS-element AR 3.2 64 bit software. In order to have an indicative estimation of the defect, an axon length/somite length ratio was calculated.

### RNA isolation, RT-PCR and gene expression analysis in zebrafish

RNA from zebrafish embryos was isolated using Trizol reagent (Invitrogen, Paisley, UK) following manufacturer’s instructions. For RT-PCR analysis following MO microinjection, RNA from about 30 embryos was isolated and 1 μg of RNA for reaction was reverse transcribed with High capacity cDNA reverse transcription kit (Applied Biosystems).

Primers used were: Fw 5′-TTGACGCGATTAACCGACTG-3′; Rv5′- GGAGAGTAAGACGGAGAGCC-3′. Individual bands were purified and sequenced.

RNA from fibroblasts and fish for qRT-PCR was extracted using the RNAeasy mini kit (Qiagen) following manufacturer instructions and then treated with DNA-free Kit (Ambion) in order to remove genomic DNA contamination. 2 μg of RNA was used to synthesize cDNA with the high capacity cDNA reverse transcription kit (Applied Biosystems). The cDNA was diluted in order to have the equivalent of 25 ng of RNA/reaction. qRT-PCR (Biorad Iight-cycler equipped with a MyIQ detection system) was performed in triplicates. qRT-PCR primers are listed in Supplementary Material, Table S4.

## Supplementary Material

Supplementary Material is available at *HMG* online.

Supplementary Data

Supplementary Data
