# Supplementary material for: Altered RNA metabolism due to a homozygous RBM7 mutation in a patient with spinal motor neuropathy
Source: Hum Mol Genet. 2016 May 18;25(14):2985–96. doi: 10.1093/hmg/ddw149 (PMC5181591; doi:10.1093/hmg/ddw149)
Supplement: Supplementary Data [file supp_25_14_2985__index.html]

Altered RNA metabolism due to a homozygous RBM7 mutation in a patient with spinal motor neuropathy — Supplementary Data 

# Altered RNA metabolism due to a homozygous *RBM7* mutation in a patient with spinal motor neuropathy

## Supplementary Data

files

- Supplementary Data - xlsx file
- Supplementary Data - docx file
