# Supplementary material for: Altered RNA metabolism due to a homozygous RBM7 mutation in a patient with spinal motor neuropathy
Source: Hum Mol Genet. 2016 May 18;25(14):2985–96. doi: 10.1093/hmg/ddw149 (PMC5181591; doi:10.1093/hmg/ddw149)
Supplement: Supplementary Data [file supp_ddw149_Supplementary_data_09052016.docx]

**Supplementary table 1.**

Whole exome sequencing in the patient detected 11 homozygous, probably damaging variants (Mutation Taster).

| **Chr** | **bp** | **ref** | **mut** | **rs** | **gene** | **MAF in ExAC** | **AA** |
| --- | --- | --- | --- | --- | --- | --- | --- |
| 1 | 20656766 | C | T | rs61746771 | VWA5B1 | 0.0018 | Q-450-* |
| 3 | 49846998 | G | - |  | UBA7 | 0 | L-689-* |
| 3 | 52552791 | T | G |  | STAB1 | . | V-1647-G |
| 5 | 115394588 | A | G |  | ARL14EPL | 0.0002 | I-135-V |
| 5 | 160973809 | A | - | rs370064343 | GABRB2 | . |  |
| 11 | 63594481 | T | C |  | C11ORF84 | . | L-339-P |
| 11 | 64799981 | C | T |  | SNX15 | 0.00001648 | R-72-C |
| 11 | 114272559 | C | G |  | RBM7 | . | P-79-R |
| 18 | 44013339 | C | T |  | RNF165 | . | T-83-I |
| 19 | 39406296 | G | C |  | SARS2 | . | P-505-A |
| 22 | 19026403 | C | T | rs9605924 | DGCR2 | 0.0004 | R-543-H |

**Supplementary table 2.**

Using Sanger sequencing, we confirmed segregation within the family for the RBM7 and the SNX15 variants.

|  | **RBM7** | **SNX15** |
| --- | --- | --- |
| Patient | G/G | T/T |
| father | G/C | T/C |
| mother | G/C | T/C |
| sister | C/C | T/C |
| sister | C/C | C/C |
| sister | G/C | T/C |
| brother | G/C | C/C |
| brother | C/C | C/C |
| brother | C/C | C/C |

**Supplementary table 3.**

The following intronic primers were used to perform sequencing of *RBM7* in genomic DNA.

| **RBM7** | **Forward** | **Reverse** | **Size** |
| --- | --- | --- | --- |
| Exon 1 | 5'-GTTTTCGTTTGTGACGCCAG-3' | 5'-TACGAAAAGAAAACGGGGTG-3' | 289 bp |
| Exon 2 | 5'-CGTTTCTGTTGTTTTTAAACTTTGTC-3' | 5'-CCCAAAATGAAACTTTAAGCTCAG-3' | 483 bp |
| Exon 3 | 5'-GGAGTGATTTCAAGTGGGGA-3' | 5'-TCATGTGTTTTGAGGCTATTCC-3' | 239 bp |
| Exon 4 | 5'-ATTCTGGCTGCATGAGAGCA-3' | 5'-TCACTTACGCCTTGAGGACT-3' | 366 bp |
| Exon 5 | 5'-TCTTTCATTTTCTGTTAACTGACCA-3' | 5'-AGCTGCTCTGTACAGTTTTTCA-3' | 543 bp |

| **RBM7** | **Forward** | **Reverse** | **Size** |
| --- | --- | --- | --- |
| Exon 1 | 5'-GTTTGTGACGCCAGGGAG -3' | 5'-CGTCACTTTCGGCCTAAACG -3' | 400 bp |
| Exon 2 | 5'-GGAAATCCGTGCATCATTTTCA -3' | 5'-CCATGTGTCAATGTTACCCGT -3' | 475 bp |
| Exon 3 | 5'- CCCGGCCAGTAGTTTGAGAT -3' | 5'-ACAACAACCCCAAAAGGCAA-3' | 360 bp |
| Exon 4 | 5'-TATTCTGGCTGCATGAGAGC-3' | 5'-CAGCCCAGTGAAAACTAAAATGA-3' | 451 bp |
| Exon 5 | 5'- TGCTTTAGTTGTGGATCCATCT -3' | 5'-TGTGACAACTTGTAAAGCTGCT -3' | 600 bp |

The following qRT-PCR primers were used to perform gene expression analysis.

| Gene | Species | | Fw primer | Rv primer |  | |
| --- | --- | --- | --- | --- | --- | --- |
| *atxn1a* | | *D.r.* | GGGTGGAAGACCTGAAAACA | GCCGAACACAAAGAAAGGAT |  | |
| *atxn1b* | | *D.r.* | TACAGACATCGCCCACAGAG | CAGCGGCACTCCTAATGCT |  | |
| *β-act* | | *D.r.* | CGAGCTGTCTTCCCATCCA | TCACCAACGTAGCTGTCTTTCTG | |  |
| *ef1α* | | *D.r.* | CTGGAGGCCAGCTCAAACAT | ATCAAGAAGAGTAGTAGTACCGCTAGCATTAC | |  |
| *HOXC6* | | *H.s.* | AAAAGAGGAAAAGCGGGAAG | CGAGGGAGAAAGGGAGAGAG | |  |
| *HOXC8* | | *H.s.* | GGGAGACGGAGAAACAGTGA | AGGTGGGAGTGTGGTGAGAG | |  |
| *HOXC9* | | *H.s.* | AGACGCTGGAACTGGAGAAG | AGGCTGGGTAGGGTTTAGGA | |  |
| *HOTAIR* | | *H.s.* | GGAGTGGGGAGTGGAGAGA | CGTGGCATTTCTGGTCTTGT | |  |
| *TUBB* | | *H.s.* | GCTGGTGGAAAACACAGATG | GTTGAGGTCCCCGTAGGTG | |  |

**Supplementary table 4 (separate file, Excel spreadsheet)**

Read counts for the up- or downregulated transcripts shared between the RBM7 and EXOSC8 patient fibroblasts.

**Supplementary Figure 1**

Conservation analysis of RBM7 orthologues show the RRM domain is highly conserved throughout vertebrates (A). Indeed, although human and zebrafish RBM7 show a low degree of homology and similarity (B), alignment of only the RRM of both proteins shows a much higher percentage of homology and similarity (C).


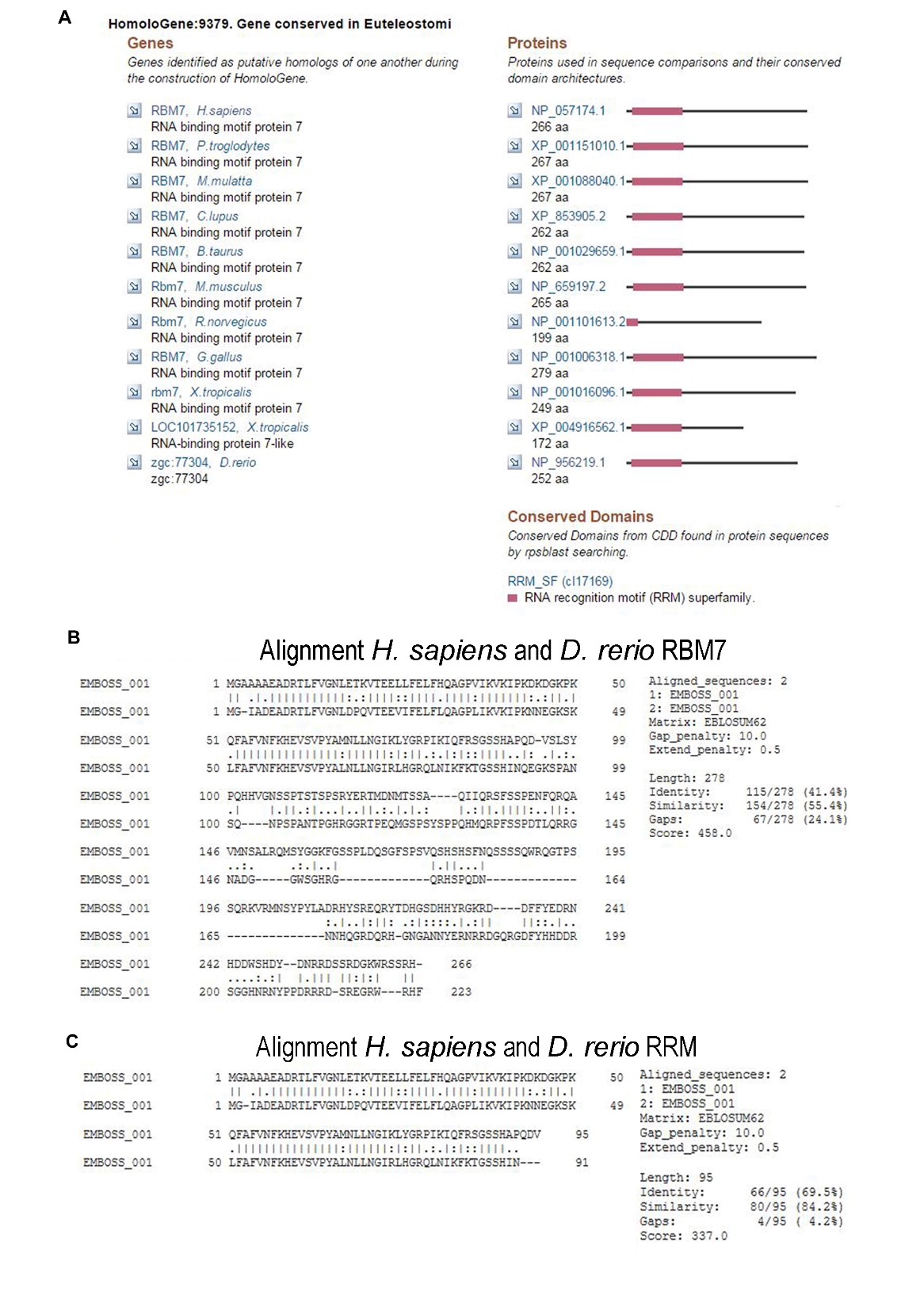


**Supplementary Figure 2**

To evaluate differences between defective and normal axons we calculated an axon length/somite length ratio (normal axons ratio =1). For each morphant type, 5 fish with a mild phenotype were randomly chosen and only the shorter axons were measured and compared to the length of the somite. n indicates number of axons analysed for each morphant. Bars indicate S.D.


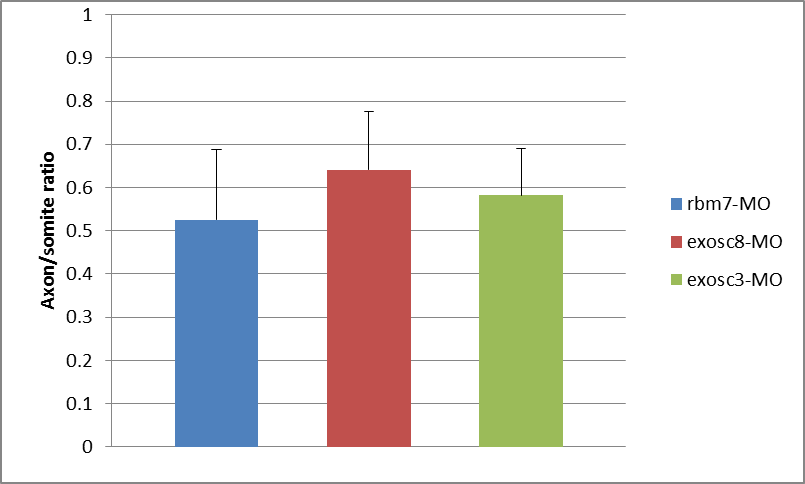


n=8

n=12

n=4
